# Care for Patients with Type 2 Diabetes in a Random Sample of Community Family Practices in Ontario, Canada

**DOI:** 10.1155/2012/734202

**Published:** 2012-07-18

**Authors:** Gina Agarwal, Janusz Kaczorowski, Steve Hanna

**Affiliations:** ^1^Department of Family Medicine, McMaster University, 175 Longwood Road South, Hamilton, Ontario, Canada L8P 0A1; ^2^Department of Family and Emergency Medicine, Doctor Sadok Besrour Chair in Family Medicine, University of Montreal, Montreal, Canada H3C 3J7; ^3^Department of Clinical Epidemiology and Biostatistics, McMaster University, Hamilton, Canada L8S 4K1

## Abstract

*Objective*. Diabetes care is an important part of family practice. Previous work indicates that diabetes management is variable. This study aimed to examine diabetes care according to best practices in one part of Ontario. *Design and Participants*. A retrospective chart audit of 96 charts from 18 physicians was conducted to examine charts regarding diabetes care during a one-year period. *Setting*. Grimsby, Ontario. *Main Outcome Measures*. Glycemic screening, control and management strategies, documentation and counselling for lifestyle habits, prevalence of comorbidities, screening for hypertension, hyperlipidemia, and use of appropriate recommended preventive medications in the charts were examined. *Results*. Mean A1c was within target (less than or equal to 7.00) in 76% of patients (ICC = −0.02), at least 4 readings per annum were taken in 75% of patients (ICC = 0.006). Nearly 2/3 of patients had been counselled about diet, more than 1/2 on exercise, and nearly all (90%) were on medication. Nearly all patients had a documented blood pressure reading and lipid profile. Over half (60%) had a record of their weight and/or BMI. *Conclusion*. Although room for improvement exists, diabetes targets were mainly reached according to recognized best practices, in keeping with international data on attainment of diabetes targets.

## 1. Introduction

Routine diabetes care remains a family practice activity for which the Canadian Diabetes Association (CDA) has set standards since 1999 [1]. Previous work in 7 Canadian provinces [2] examining the management of T2DM patients between 1998 and 1999 found family physicians were falling short of best practice guidelines, particularly in screening for microvascular disease, managing hypertension, hyperlipidemia, and prescribing antiplatelet medication. Currently, family physicians' performance levels in their regular patients in Ontario are unknown. This knowledge could help family physicians target certain areas of diabetes care, in service planning for the future.

The goals of this study were to examine a small group of actual family physicians' management practices in following best practices and achieving targets in actual clinical care following on from the widespread dissemination of best practice documents, in the following areas: glycemic screening, control and management strategies, documentation and counselling for lifestyle habits, prevalence of comorbidities, screening for hypertension, hyperlipidemia, and use of appropriate recommended preventive medications (see the appendix). Specific outcomes are described in Table 1. This paper describes the results of a detailed chart audit looking at diabetes care in the practices of 18 community-based family physicians in Ontario.

## 2. Methods

We conducted a retrospective chart audit in a total of 18 family doctors' offices in Ontario as part of a study evaluating the effectiveness of a community-based diabetes risk-assessment program (the results of which are described elsewhere). The practices were predominantly fee-for-service and some primary care group practices with a mixture of fee-for-service and capitation. Practices provided lists of names of all patients who were rostered to each family physician and who were over the age of 40, for invitation to the diabetes-risk assessments. From these, a number of charts of people with diagnosed diabetes were unintentionally randomly selected. Patients who had a previous diagnosis of diabetes prior to February 2004 were audited and information concerning routine care that they received between February 1st 2004 and February 1st 2000 extracted. This care would have been received before the diabetes risk-assessment program started and thus would not have been affected by it.

The chart auditors consisted of 2 trained health research assistants. The data extracted was based upon the most recent and relevant best practice guidelines [1, 3]. Where there were several readings, these were averaged for the whole year and the average reading was considered in the analysis. Where data were not available in the chart it was assumed those actions were not done and therefore missing. Physician demographic data (gender, years in practice, and certificant status with the College of Family Physicians of Canada) and practice demographics were collected by a direct questionnaire to each physician. Data was double-entered into a database.

It was estimated that for a sample size of approximately 100, under the most conservative assumptions, the 95% confidence intervals could be calculated to the nearest +/− 10% [4]. Analysis of the data was conducted using SPSS [5]. Physician and patient demographic data were summarised using descriptive statistics. Data was analysed at the patient level. Univariate analysis was performed to look at the proportions of patients who had certain clinical characteristics. To take into account clustering due to the potential similarities of patients attending a particular physician [4], the intracluster correlations (ICCs) and their 95% confidence intervals were calculated.

## 3. Results

Family physicians' characteristics are shown in Table 2. A total of 96 charts were audited, of people who had a diagnosis of diabetes recorded in their charts (see Table 3 for characteristics). The sample consisted of 51% males, 1/3 employed, 38% retired and the mean age was 68 years old (SD = 12.02). The mean number of years since diagnosis of diabetes was 8.8 and all patients had visited their family doctor at least once during the 1 year audit period. Patients were unevenly clustered between the family physicians, and numbers of patients with diabetes per physician ranged from one to eleven.

### 3.1. Glycemic Control, Monitoring, and Management

The average A1c reading in this group of patients was 7.07; mean A1c was within target (≤7.00) in 76% of patients (ICC = −0.02) (Table 4). Ninety-six per cent of patients had one A1c reading during the 1-year audit; 75% had four A1c readings in the year (ICC = 0.006).

A minority of patients were treated with lifestyle (12.5%). Most (89%) were on medication; 42% on oral medication, about half just one oral agent (50%), a small proportion on insulin only (10%), and very small proportion on both types of agent (1%). In more than half of the charts exercise (57.3%) and diet (57.7%) were mentioned as having been discussed, and a small number (15.5%) had been referred to a dietician.

### 3.2. Adherence to Best Practice Guidelines

Secondary data analysis shows that 17.8% of patients (*n* = 16, ICC = 0) had an A1c less than or equal to 0.06%, and 32.2% (*n* = 29, ICC = 0) had achieved an A1c of less than or equal to 0.07% and 50.0% (*n* = 45, ICC = 0) had an A1c greater than 0.07%.

Optimal control for those at risk of nephropathy (<0.065%) was achieved in 27.5%, optimal control in general was achieved in 23.1%, and suboptimal control was achieved in 49%.

### 3.3. Prevalence of Diabetes Related Co-Morbidities and Complications

More than half of the patients had hypertension (59%) a quarter had dyslipidemia (24%), and 15% had diabetes related complications (Table 5).

### 3.4. Screening for and Management of Risk Factors

Nearly all patients had documentation of blood pressure readings (98%) and lipid profiles (97%) (Table 6). The mean BP reading was 134/74 mmHg and 31% (ICC = 0) met the target for systolic and 85% (ICC = 0) met the target for diastolic BP. The average number of BP readings was 15 (ICC = 0.054). The mean total cholesterol was 4.58% (ICC = −0.067). Lipid-lowering agents were prescribed for 39% (ICC = 0.382). ASA was prescribed for 23% (ICC = 0.0456). Forty per cent were prescribed an ACE I inhibitor (ICC = −0.023).

### 3.5. Lifestyle Habits

BMI could not be calculated for 40% (ICC = 0.094) (Table 7). Where BMI was recorded, 27% were obese and 20% were overweight (ICC = 0.132). One in 10 were current smokers (10.2%, ICC = 0.433) and only 1% consumed alcohol heavily (ICC = −0.150).

## 4. Interpretation

This study presents data from medical charts covering 18 different nonacademic practitioners in a small region of Ontario. Existing Canadian-published data concerned with management and control of diabetes in family practice concentrates on small numbers of practices [6, 7] or has looked at recruiting charts of those who have attended the physician for checkups, and have not been randomly selected [8] therefore may not be an accurate representation of the state of diabetes management in Canada. Others use different time periods for audit of charts and different criteria for physician and patient recruitment, and focus on different locations [8, 9]. In this study in the Grimsby region, family physicians were successfully monitoring A1c levels, and reaching targets in 3/4 of patients. Nearly 2/3 of patients had been counselled about diet in some form, more than 1/2 on exercise and nearly all (90%) were on medication. Nearly all patients had a documented blood pressure reading and lipid profile. Over half (60%) had a record of their weight and/or BMI.

In a previous and much larger Canadian study, Harris et al. [2] collected data from 16 practices, 55 physicians, 549 charts, and 10 charts per physician, located all over Canada for one year from February 1st 1998. Less patients had reached CDA targets for A1c (40% less, with A1c target reached in 35%) and fewer had their A1c readings recorded (25% less); 25% more patients were on oral medication only; nearly 20% less of the charts mentioned dietician counselling and nearly 30% less charts mentioned exercise; 30% less charts had lipid profiles measured and the mean total cholesterol was higher; there was more hypertension and hyperlipidemia. These results suggest that the Grimsby group of family physicians have been more successful in the care of their patients than those in the Harris's study conducted 7-8 years earlier. This may be indicative of a wider change amongst family physicians and their behaviour in practice.

A more recent study in Newfoundland, Canada, in 160 patients from 8 practices, [7] showed that 48% had reached best practice guideline targets for A1c. More patients were at target for systolic and diastolic BP readings, and the same percentage were documented as having hypertension, but more as having cerebrovascular and coronary artery disease. It was not possible to compare medication, lifestyle and physician demographics since these were not reported. When physicians were asked to estimate what proportion of patients were at targets for A1c, this group of practitioners overestimated their results by 20%. Forty percent were at target for systolic BP compared to 31% in Grimsby and 42% for diastolic BP compared to 85%.

It is possible that internationally, diabetes targets are improving in many geographical areas. A large study in Nijmegen, Netherlands, looked at the progress of diabetes targets between 1993 and 1999 in a network of academic family practices that were informed of their own clinical practice using feedback from audits. They concluded that over the 7-year period, A1c targets of <7.0 mmol/L were met by 22% more patients, lipid targets by 20% more patients, and that 11% more met blood pressure targets [10]. Another North American study compared attainment of targets in a cohort of 86 patients over a 4-year period in academic practices, from 1999 to 2003 and concluded that though A1c values improved, actual target attainment overall did not change [11]. In our study, 76% achieved A1c targets and extrapolating from Harris's data it is possible that an improvement from 35% to 76% was achieved for attainment for A1c targets over a 7-8-year period.

Improvement of targets may partly be due to change in practice structure and feedback that has and is occurring. A recent study across North America of 141 clinicians and 822 patients concluded that certain practice-based factors worsened A1c outcomes in patients; for example patients in academic practices, in multispecialty practices or in solo practitioners' practices had worse A1c outcomes; whereas patients in practices having a nurse practitioner or physician assistant had improved A1c outcomes [12]. They point out that in primary care their sample of patients were complex with advanced disease and complications, and 40.5% achieved A1c levels of <7 mmol/L. They recommend that diabetes registries, and specific clinical parameters for disease monitoring are required. Other evidence also shows that audit feedback can improve patient outcomes and target attainment [12, 13], and may result in more frequent monitoring of patients [14]. The audit presented here is an example of such an audit that can be easily set up and disseminated to practitioners or that they can set up themselves in an electronic medical record.

Examining attainment of diabetes targets internationally, Canadian practitioners' achievement of 76% of patients at target for A1c compares favourably; in Estonia, a study of 200 patients in primary care showed 50% had achieved their targets A1c levels of <7 mmol/L [15]; in Singapore where care has been traditionally in endocrinology clinics but is being shifted to primary care, after this shift of care, more than 50% achieved their A1c targets in primary care compared to endocrinology clinics [16]; in Belgium in 120 GP practices 54% achieved their A1c levels of <7 mmol/L [17]. It should be mentioned that targets vary in countries, though as far as possible a target of <7 mmol/L or <7 mmol/L has been quoted. Also, in international situations, certain practice-based factors will be prevalent that can affect clinical care. However, these global figures provide a crude estimate upon which to compare standards of care.

In this data, reported ICCs are consistent with the literature [18, 19] with higher values for process issues and are thus more likely to reflect a particular practicing-style of a particular family doctor. Though the ICC values are small, they are positive, and have small confidence intervals, indicating that for certain variables such as mean FPG value (ICC = 0.16), being on medication/insulin for glycemic control (ICC = 0.05), on lipid lowering medication (ICC = 0.02), numbers of readings for blood pressure (ICC = 0.06), and dietician consultation having been sought (ICC = 0.08), the specific physician to which the patient belonged would somewhat influence the patients' values for these variables. Some ICC values were zero or near zero, indicating that the physician a patient belonged to did not exert any influence on that variable [20].

Remuneration methods of each practice could have affected the diabetes care offered in that practice. In capitated models of remuneration there are incentives for reaching certain targets for patient care, but these do not exist in a fee-for-service model of care. It was not possible to explore this effect in this data due to the small number of practices. Typical clinical practices of each physician with respect to diabetes care were not collected due to the small number of physicians (*n* = 18) in the sample, though the area sampled had a mixture of mainly fee-for-service practices and a few groups paid on a capitated model.

**Limitations of This Study**

Though the results may not be generalisable to practices from different areas, or directly to international figures, they will be comparable to those of similar demographic make-up. In 2001, compared to Ontario as a whole, the Grimsby area population had less ethnic minorities, was more educated, and had a higher income [21]. Although diabetes care seems to be improving it is possible that this improvement is just regional (not Ontario-wide or Canada-wide) and specific to the Grimsby area.

**Implications and Conclusion**

Target attainment was comparable to other information available from Ontario. It may not be feasible to suppose that further improvements can be made to care and realistically, this may be as good as clinical care can get in primary care for diabetes. Comparison with international data seems to imply that there is global improvement in diabetes targets, with some geographical areas improving more than others. Challenges to improving care further have been “clinical inertia” [22], failure to intensify therapy appropriately [2] and difficulty in keeping up to date with guidelines [23]. Care can be improved by continuous audit and feedback to practitioners [12]. However, despite these challenges, these results from this audit show that although there was room for improvement, diabetes targets were mainly being met according to the best practice guidelines in this sample of family doctors and this population.

## Figures and Tables

**Table 1:** Outcomes used.

	Outcome description	Correlates with CDA guideline number (1)
Primary outcome	(1) Percentage of patients in which glycosylated haemoglobin (A1c) targets were reached (≤7.00)	1

Secondary outcomes	(1) Percentage of patients who reached the following glycemic targets; at least 4 A1c readings in a 1-year period and a mean fasting plasma glucose (FPG) within target of 4.0–7.0 mmol/L	1
	(2) Percentage of patients who were managed by lifestyle modification as documented in their chart and other descriptive information about glycemic management	5
	(3) Percentage of patients exercising regularly as documented in their chart and other descriptive information about diet and exercise	4
	(4) Prevalence of comorbidities (hypertension, dyslipidemia, retinopathy/neuropathy/nephropathy, cerebrovascular disease, coronary artery disease, and specialist consultations	
	(5) Percentage of patients reaching blood pressure targets	3
	(6) Percentage of patients who had lipids monitored	2
	(7) Percentage of patients who were prescribed aspirin and ACE inhibitor medication	8 and 9
	(8) Percentage of patients in whom there was documentation of smoking and alcohol consumption status	6 and 7
	(9) Percentage of patients in whom there was documentation of body mass index	

**Table 2:** Physician characteristics reported in summary.

Characteristic (*N* = 17)	Value (median; SD)	Range
Female gender (*n* = 16)	26%	
Years since graduation	17.4 (17.0, 8.6)	4–30
Recent graduate (<5 years) (*n* = 16)	12%	
Certificant of CFPC (*n* = 16)	88%	
Canadian medical graduate (*n* = 16)	82%	
Local medical graduate (McMaster) (*n* = 16)	41%	
Foreign medical graduate	18% (*n* = 16)	
How many years in practice (self-reported)? (*n* = 9)	10.9 (10; 8.21)	0.5–24.0
Approx. how many pts. do you have in your practice (self-reported)? (*n* = 9)	1472 (1500, 601)	700–2500
About how many of your pts. are 40 yrs or older (self-reported)? (*n* = 9)	785 (600; 417)	420–1500
Estimate what % of your pts. 40+ have been diagnosed with diabetes (self-reported) (*n* = 9)	11.4 (8.5; 10.5)	2–30

**Table 3:** Patient characteristics.

Characteristic (*N*)	Value (median; SD)	Range
Mean age at diagnosis in years		
Mean duration of diabetes in years (81)	6.8 (7.0)	0.6–42.2
Mean age at audit in years	68.0 (69.5, 12.0)	42.6–89.4
Female (96) in %	49	

Employment status	Percent (*n* = 96)	

Employed	29.5	
Unemployed	5.3	
Retired	37.9	
On disability	3.2	
Not reported	24.2	

Age category	Percent (*n* = 96)	

<39	4.2	
40–44	3.1	
45–49	8.3	
50–54	13.5	
55–59	10.4	
60–64	15.6	
65–69	14.6	
70–74	8.3	
75–79	15.6	
80–84	6.3	
85–89	4.2	
>90	3.1	

Years since diagnosis of diabetes (*n* = 81)	8.8 (7.0)	2.2–44.2

**Table 4:** Glycemic control, monitoring, and management.

Measure	Value	Median, SD, Range (where applicable)	ICC (95% confidence interval)
Glycemic screening and control			
A1c tested once, % of patients	96.00		0.00 (−0.003311, 0.003311)
Mean A1c, %	7.07	7.00, 1.1, 5.0–11.0	0.00 (−0.003311, 0.003311)
Mean A1c within target of ≤7.0, %	76.0		−0.018989 (−0.021702, −0.016276)
Within target of at least 4 A1c readings in 1 year period, % patients	75.3		0.057496 (0.037328, 0.077664)
FPG tested, % patients	85.00		0.00 (−0.003311, 0.003311)
Mean FPG, mmol/L	7.84	7.58, 1.94, 3.90–13.10	0.163798 (0.117942, 0.209654)
Mean FPG within target of 4.0–7.0, %	46.4		0.00 (−0.003311, 0.003311)
RPG tested, % patients	43.80		
Mean RPG, mmol/L	8.40	7.83, 2.86, 2.30–17.40	0.0787675 (0.0734361, 0.0840989)

Glycemic management strategies			
Lifestyle only, % patients	0		
Lifestyle at all, % patients	12.5		−0.02244 (−0.026272, −0.018608)
Medication	89.00		0.019339 (0.010126, 0.028552)
Oral agents only, % patients	42.0		
Of patients on oral agents:			
1 oral agent, % patients	50.0		0.013274 (0.005886, 0.020662)
2 oral agents, % patients	43.0		−0.02424 (−0.028659, −0.019821)
3 oral agents, % patients	7.4		−0.10593 (−0.139121, −0.072739)
Insulin only, % patients	10.4		0.048603 (0.030906, 0.066300)
Insulin + oral agents, % patients	1.0		−0.09307 (−0.121453, −0.064687)

Counselling			
Dietician, % patients	15.5		0.07923 (0.053232, 0.105228)
Chart mentions diet counselling, % patients	57.7		0.00 (−0.003311, 0.003311)
Dietician and/or chart mentions diet counselling, % patients	62.0		0.098409 (0.067513, 0.129305)
Exercise, % patients	57.3 (mentioned)		0.066321 (0.043750, 0.088892)

**Table 5:** Prevalence of diabetes-related comorbidities and complications.

Diagnosis	% of Patients	ICC
Hypertension	59.8	0.158733 (0.113940, 0.203526)
Dyslipidemia	24	−0.0171 (−0.019203, −0.014997)
Retinopathy, nephropathy, or neuropathy	15.4	0.0572 (0.037114, 0.077286)
Cerebrovascular disease	2.1	0.005977 (0.000816, 0.011138)
Coronary artery disease	7.2	−0.081 (−0.104965, −0.057035)
Cardiovascular consultation	23.7	−0.03058 (−0.037081, −0.024079)
Endocrinology consultation	5.2	−0.07619 (−0.098420, −0.053960)

**Table 6:** Screening for and management of risk factors.

Clinical activity	Value	Median, SD, range	ICC
		(where applicable)	
BP result documented, %	98		
No. of BP readings	15	15, 9, 0–24	0.06424 (0.042231, 0.086249)

Mean BP;			
Systolic mmHg	134	135, 12, 102–165	−0.14068 (−0.187383, −0.093977)
Within systolic BP targets of ≤130	31.0		0.00 (−0.003311, 0.003311)
Diastolic, mmHg	74	74, 7, 54–94	0.03471 (0.020974, 0.048446)
Within diastolic BP targets of ≤80	84.5		0.00 (−0.003311, 0.003311)
Lipid profile obtained once, %	97		0.040813 (0.025322, 0.056304)
Mean total cholesterol	4.58	4.49, 0.99, 2.43–7.98	−0.06611 (−0.084751, −0.047469)

Medications to prevent complications	% Patients		
On ACE I	40.0		−0.02382 (−0.028101, −0.019539)
On lipid lowering medications	39.2		0.38283 (0.306461, 0.459199)
On ASA	22.7		0.045959 (0.029007, 0.062911)

**Table 7:** Lifestyle habits.

	% Patients	ICC
Current smokers	10.2	0.433308 (0.354187, 0.512429)
Alcoholic	1.0	−0.15016 (−0.200681, −0.099639)
Occasional/social alcohol	25.5	
BMI obese >30	26.5	0.132369 (0.093368, 0.171370)
BMI overweight 25–30	20.4	
BMI not possible to calculate from chart	39.8	0.093952 (0.064173, 0.123731)

**Table 8:** 

	A1C (%)	FPG/preprandial PG (mmol/L)	2-hour postprandial PG (mmol/L)
Target for most patients	≤7.0	4.0–7.0	5.0–10.0
Normal range (consider for patients in whom it can be achieved safely)	≤6.0	4.0–6.0	5.0–8.0

**Table 9:** 

Risk level	LDL-C (mmol/L)	TC: HDL-C
High (most patients with diabetes)	<2.5 and	<4.0

**Table 10:** 

Indications for treatment: BP >130 mm Hg or >80 mm Hg despite
lifestyle modification
Target: BP *⩽* 130/80 mm Hg

**Table 11:** 

Priority of clinical issue	Target population	Interventions
Vascular protection	All people with diabetes	ACE inhibitor (as indicated)
		Antiplatelet therapy (80–325 mg/d ASA)
		Blood pressure control
		Glycemic control
		Lifestyle modification: nutrition therapy,
		regular physical activity, weight management
		Lipids control
		Smoking cessation

## Appendix

### Extract from the CDA 2003 and 2008 Guidelines

1. A1c: Monitored every 3 months as per CDA guidelines. Targets should be as shown in Table 8.
2. Lipids: To be monitored. Targets for lipids should be as shown in Table 9.
3. BP: To be monitored. Targets should be as shown in Table 10
4. Everyone should do exercise
5. Everyone needs lifestyle modification
6. Be a nonsmoker
7. Stay within healthy drinking guidelines: limit intake to 1-2 drinks/day (<14 standard drinks/week for men and <9/week for women). Also, vascular protection should be on the following:
8. ACE inhibitor;
9. ASA (see Table 11).

*Note*. 2008 guidelines differed from the above in the following ways:

1. A1c target of ≤6.5 should be considered in those in whom it is reasonably safe to do so;
2. LDL-C target for most patients with diabetes should be <2 mmol/L (ratio same);
3. exercise of 150 minutes vigorous activity per week recommended;
4. For vascular protection ARB is recommended.
